# Society Proceedings

**Published:** 1899-03

**Authors:** 


### SOCIETY PROCEEDINGS.

### IOWA STATE VETERINARY MEDICAL ASSOCIATION.

THE eleventh annual meeting was called to order at Des Moines, January 10, 1899, at 10 A.M., by the Secretary. There being neither President nor Vice-Presidents present, Dr. D. H. Miller, of Harlan, was made President *pro tem.* Instead of the usual roll-call, those present were asked to write their names on a register provided for the purpose. Later on the register was found to contain the signatures of members present, as follows: Drs. P. O. Koto, Forest City; S. T. Miller, Shelby; H. E. Talbot, Des Moines; D. H. Miller, Harlan; A. S. Brodie, Cedar Falls; G. E. Noble, Osage; M. Stalker, Ames; S. Whitbeck, Decorah; J. I. Gibson, Denison; H. Shipley, Sheldon; W. B. Niles, Ames; S. H. Kingery, Creston; S. K. Hazlet, Oelwein; J. G. Parslow, Shenandoah; John E. Brown, Oskaloosa; P. Malcolm, New Hampton; S. H. Johnston, Carroll; R. R. Hammond, Le Mars; William Drinkwater, Monticello; A. A. Peters, Winterset; H. L. Stewart, Oakley; C. E. Stewart, Chariton; W. H. Austin, Newton; A. T. Peters, Lincoln, Neb.; J. A. Campbell, Des Moines; J. F. Kennedy, Des Moines. The following-named visitors were also present: F. J. Neiman, Marshalltown; C. W. Stevens, Knoxville; H. E. Titus, Maxwell; H. S. Titus, Rhodes; G. E. Noble, Osage; Senator J. M. Emmert, Atlantic; —— Yeoman, Des Moines. The minutes of the last annual meeting were read and approved. Drs. Shipley and Talbot were appointed to fill vacancies on the Board of Censors.

The Treasurer rendered a report showing all bills paid to date and $28.83 in the treasury. Drs. Whitbeck, Koto, and Brodie were appointed as an auditing committee, and the Treasurer's report was referred to them.

The Secretary's report was read, being in part a review of the life-history of the organization. It said, in part: "Organized in 1887, with sixteen charter members, it began a sort of 'home mission' effort. Article 2 of our Constitution says: 'The objects of this Association are the mutual advancement of its members in veterinary science, the cultivation of fraternity, and the elevation of the veterinary profession.' What could be of greater interest to the veterinarians of Iowa?—'mutual advancement in veterinary science.' Let the watchword be upward and onward, and by united effort we will advance. 'Cultivation of fraternity,' meaning a more intimate acquaintance, a closer relationship, a brotherhood, a kindliness of feeling one for another, coöperation in all things pertaining to the advancement of the profession's interest, and 'elevation of the profession.' As to the Association's influence along these lines there can be no doubt. It would be impossible for any wideawake, observing student of veterinary science to attend one of our meetings and not receive some new light. Our meetings have excited the most complimentary notices from veterinarians in almost every State in the Union. As to what the Association has done toward 'elevating the profession:' Do we believe that through the Association we have made any gains along that line? Is there a man here who has been practising the veterinary profession for ten years past in Iowa who does not believe that the profession has a higher standing with the general public to-day than it had ten years ago? and have the influences that have gone out from these meetings in one way and another played any part in bringing about such a change? Have the joint sessions that we have held with the members of the State Agricultural Society, and the more intimate relationships between veterinarians and stockmen thus resulting, played any part? In Iowa we now have about two hundred graduated veterinarians. With the increase of veterinarians in the State we have not kept up a corresponding increase in our membership. Why is this the case? I know that the veterinarians of this Western country during the past few years have, to put it plainly, been *hard up*, and many of them have answered my letters saying their practice would not justify the expenditure of a sufficient amount of money to attend the meetings. The one thing needed above all others, if it were possible, would be to issue printed reports of our meetings. Last year arrangements were made whereby the report might have been published in the report of the State Agricultural Society, but two of the papers were not turned over to me at the time of the meeting, and I failed to get them in time to go in. The report of our meeting of two years ago was to have been printed by the publishers of the *Veterinary Magazine*, but that publication suspended before our proceedings got into print. After this meeting we will have the notes and papers of the last three meetings on hand, which should by all means be published." Discussions then followed on subjects pertaining to the interests of the Association and the veterinary profession, in which Drs. Brodie, Whitbeck, Gibson, and others took part, the impression prevailing that the veterinarians generally appreciate the value of the Association, and that the membership and attendance at the meetings will increase as the price of live-stock increases and business improves. On vote, the report was accepted.

The Board of Censors reported favorably on the applications for membership of G. E. Noble, D.V.S., Osage, Ia.; H. E. Titus, D.V.M., Maxwell, Ia.; F. J. Neiman, V.S., Marshalltown, Ia.; C. W. Stevens, M.D.C., Knoxville, Ia. On vote, all were duly elected to membership.

The meeting then adjourned until 1.30 P.M.

*Afternoon Session, 1.30 o'clock.*—Meeting called to order by President Johnston.

The Auditing Committee reported having examined the Treasurer's accounts and found the statement as rendered to be correct. The report was then accepted and the committee discharged.

President Johnston then addressed the meeting as follows: "It is with pleasure I greet you. Another year of our Association's life has passed into history—a year of marked progress in veterinary science—and only those interested in the work know what marked advantages the present veterinarian has over the past. It is the duty of every veterinarian to educate, as far as possible, those interested in the benefits to be derived from veterinary science rather than work in that mysterious way so commonly met with among the uneducated in all professions to some extent, but especially so in ours. The assembling together of so many different minds cannot help but result beneficially, but the veterinarian should always remember that the 'man makes the profession,' not the profession makes the man. He should be of exemplary habits—upright, honorable, and just in all his dealings between his employer and himself. It is with regret that I see the deplorable results of ignorance as displayed through lack of veterinary education in the United States Army, when millions of dollars' worth of property are left to the care of uneducated veterinarians, the sufferings of the bravest animals in war and the most docile in peace being unnecessarily increased. The Iowa State Veterinary Medical Association, of which we have a right to feel proud, came into life in the year 1887, at Ames, Iowa, with sixteen charter members. Dr. A. B. Morse, of Des Moines, was its first President, and Dr. J. J. Street, of Ames, was Secretary. In the past ten years we have enrolled one hundred and forty-five members, some of whom have left the State and others have been suspended for non-payment of dues, leaving our present organization with about seventy-five members in good standing. There should be no excuse now. The past few years were not very remunerative to veterinarians, but the present prospects are much brighter. It is unnecessary for me to dwell on the condition of our State laws. We should make united efforts to improve them. Let each member seek to so educate his legislators that they may understand that it is to benefit the general public, and not for party or class pecuniary motive, that we are working; then I will have no doubt of the results. It is encouraging to note that the medical profession is awakening to the realization of the transmission of disease through diseased meat and milk, and we hope and trust that very soon all abattoirs and dairies will be operated under inspection and supervision of qualified veterinarians. Under the campaign of our veterinary and medical journals, assisted by the public press, good results should follow in the near future. Our programme for this meeting has been selected with great care and diligence on the part of our Secretary, and it is for you, gentlemen, to make the meeting the success it should be by freely participating in the discussions. By so doing we will go home feeling that we have been well repaid for our efforts."

The reading of correspondence was then called for. The Secretary read a communication from the Missouri Valley Veterinary Association, through its Secretary, inviting the members of the Iowa State Veterinary Medical Association to become members of their Association. The invitation was duly appreciated, but it was apparently the sense of the meeting that as a body we had no authority to take any action further than to leave it with the members to do as they might see fit individually.

A letter was also read from Dr. D. E. Salmon, chairman of the Committee on United States Army Legislation of the American Veterinary Medical Association, soliciting aid for that cause. The members were requested to do what they could personally, and a committee was appointed to draft suitable resolutions. The members of the Committee on Resolutions were Drs. Niles, Whitbeck, and Parslow.

The report of the Committee on Sanitation was called for. Dr. Stewart was the only member of the committee present, and he had no report to offer. This being a very important subject, it was decided to devote some time to discussion thereon. Dr. Niles thinks that veterinarians generally have not given the attention to the subject they should. He thinks they should be sufficiently well posted to give intelligent advice to farmers and others on such subjects, and a special effort made to educate the public along the line of general sanitation. He thinks the easiest and best way to control contagious disease is by quarantine measures. He thinks "prevention is better than cure." Dr. Johnston thinks this the only means of controlling contagious diseases, and farmers should be so taught. Dr. Miller says we need more and better laws on the subject; that farmers are not inclined to take sanitary advice, and there should be laws to enforce it. Dr. Brown thinks the laws we already have should be enforced. We have a State law prohibiting any traffic whatever in dead hogs—hogs that have died of any disease or been killed on account of any disease, and yet they are hauled over the public highways to rendering establishments. The same State law provides that dead hogs shall either be burned or buried three feet below the surface. We go through the country and find these dead hogs piled up in the fence-corners or lying in the fields where they dropped, decaying, and the germs of disease being washed down into streams or carried by dogs, birds, or other means over the country; and still we cry for more law and talk about controlling hog-cholera while this sort of thing exists all over the State. Dr. Whitbeck thinks hog-cholera can be controlled by proper quarantine regulations, and advocates a State law giving veterinarians authority to issue quarantine. Dr. Austin says one of the worst features with which he comes in contact is the persistent feeding of dead stock to livestock, as practised by the Germans, especially of his locality.

Dr. Niles reported for Committee on Disease and Treatment.

Discussions followed on "Parturient Apoplexy and Dr. Schmit's Treatment with Potassium Iodide." Dr. Talbot had tried it twice; both cases died. Others had tried it with mixed results.

In regard to the "Diarrhœa of Calves," Drs. Niles, Drinkwater, Austin, Johnston, and others thought it followed only where the cows had been highly fed. Dr. Niles had not seen this disease in grass-fed cattle. Dr. Brodie asked if Dr. Niles did not think that the disease came as an infection through the umbilical cord. Dr. Niles thought not in this form. Dr. Shipley thought it might be due to an entire change in food during later pregnancy. Dr. Hammond saw three herds—twenty cows in each herd; three or four calves that came first were healthy, after that all the calves died.

Dr. Whitbeck spoke of the "Hard Growths which form after Castration in Pigs." Dr. Johnston says it is an adhesion of the divided cord to wounds in the scrotum; removes with knife. Dr. Stewart thinks these adhesions are the result of the cord being divided too close to the testicle; compared it with castration of mules; says that the cord is longer in mules than in horses, and more of it should be taken off. Dr. Shipley thinks the trouble is sometimes due to the incision being too small and too high. Dr. Austin castrates sheep with an emasculator; cuts off the bottom of the scrotum and both seeds at once with the instrument. Dr. Shipley asked about "Sheep Dips." The Fort Collins lime and sulphur dip was recommended. Dr. Talbot reported good results in a large flock by four dippings with a solution of chloro-naptholeum. "On the Use of Cathartics," Dr. S. Whitbeck reports good results from the use of from ℥iv to ℥vi of aloes made into a bolus with spirits terebinth., administered per rectum; empty rectum by enema, then insert ball as far as we can reach; gets an action in from two to six hours, usually about four hours. Dr. Johnston has had good results in this way. Dr. Miller uses a capsule. Dr. Shipley uses large doses of nux vomica in these cases, administered per rectum.

Dr. Niles reported for Committee on Speakers for Institutes, saying that several speakers had been placed, but that the institute season was well advanced before the work was undertaken. The report was accepted and the committee continued another year.

The meeting then adjourned until 8.30 P.M.

*Evening Session*, 8.30 *o'clock*.—Joint meeting of the Iowa State Veterinary Medical Association and Iowa State Agricultural Society was called to order by President Johnston.

Dr. W. B. Niles was introduced and read a paper on "The Practicability of Serum-therapy in Hog-cholera."[1] Discussion was then indulged in. Dr. McBirney was called for, giving some of his experiences in using the treatment in Page County. He said that, on the whole, the results were more satisfactory than last year; that it had been the means of saving about 80 per cent. Dr. Stalker asked how the comparison was arrived at between treated and untreated stock. Dr. McBirney replied that the method is to leave an equal number of both diseased and healthy animals untreated. It was only used in diseased herds. Dr. Niles saw two herds in adjoining fields, equally exposed and under equal conditions. The treated herd fared much better than the untreated one. Dr. Peters, of Lincoln, Neb., said they had treated about 5000 head, 76 per cent. of which were sick, and they had saved 80 per cent. of all; used as "check" healthy animals of the same herd. Of these over 80 per cent. died. Much more is known of serum to-day than ever before. The serum we are using now is much stronger and better. Serum will be practical as a treatment for hog-cholera. Mr. Wallace, editor of *The Farmer*, thinks the only way to get rid of hog-cholera is through quarantine, isolation, etc. Dr. Stalker says with proper authority and management, giving township trustees absolute control and authority to quarantine and dispose of sick hogs, $80,000 will stamp out the disease.

Dr. J. M. Emmert, late member of the State Board of Health, and now State Senator from Cass County, was introduced and read a paper on "A Campaign of Education."[2] The paper was much enjoyed by all present, and discussion again followed. Dr. Gibson said one subject which is deserving of some special thought is infectious catarrhal pneumonia in cattle. It is becoming something of a scourge in Iowa, and it may yet require some special attention from our Legislature to cope with it. It seems to be working its way westward from Eastern centres. He also spoke on the contagiousness and spread of tuberculosis as he had observed it while serving in the capacity of State veterinarian, giving several illustrations. Dr. Stalker reported a case where he was called to examine the cattle and found several of them to be tuberculous. He also found that in the family that had used milk from these cattle five young people had recently died of consumption. Investigation failed to show that the ancestors of these young folks had ever been afflicted with the disease.

The meeting then adjourned to meet for the clinical part of the programme at Dr. Talbot's office next morning at 9 o'clock.

*Wednesday*, 11th, 9 A.M., at Dr. Talbot's office.

The members gathered and spent the forenoon witnessing the clinical

[1] See page 151.

[2] See page 145.

part of the programme. This was a new feature in our meetings, and was much enjoyed by all present.

The cases operated on and the operators were as follows: Cryptorchid (single), by Dr. C. E. Stewart, Chariton; coccic curvature, by Dr. H. Shipley, Sheldon; spavin (ligamentous operation), by Dr. W. B. Niles, Ames; string-halt (division of lateral extensor of phalanges), by Dr. J. E. Brown, Oskaloosa; hernia (ventral, radical operation), by Dr. S. Whitbeck, Decorah; quittor, by Dr. S. H. Johnston, Carrol. Two or three other cases that were to have been operated upon failed to arrive on time. The operators all acquitted themselves in fine style. The members lost no opportunity to discuss methods of operating among themselves, and a most profitable session was thus spent.

*Wednesday Afternoon,* 1.30. The members having reassembled at the room, the meeting was called to order by President Johnston.

Dr. S. H. Kingery was called upon and read a paper, the title of which was "Interesting Notes from Cases in Practice." Discussion followed. Drs. Stewart and Brown each reported cases in which corn-cobs had become wedged in the upper molars, so the animals could not eat. Dr. Whitbeck reported being called to examine a case that "would not eat." On investigation he found the halter so small that the horse could not get his mouth open. Dr. Stewart treats choking by pumping warm water into the œsophagus. Mr. Yeoman, of the *Spirit of the West*, was present, and spoke of having had a mare that sustained a rupture of the vagina while foaling. Under treatment she made a perfect recovery. Would it be advisable to breed again? Drs. Johnston, Shipley, Stewart, Stevens, and others spoke on the subject, and it was generally admitted that such cases are generally susceptible of successful treatment, and that such cases might be rebred with impunity after complete recovery had taken place.

Dr. D. H. Miller, of Harlan, Iowa, was then introduced and read a paper on "Surgical Interference with Actinomycotic Growths." Discussion followed. Dr. Parslow thinks many of the cases in which abscesses form around the throat are cattle-distemper, not actinomycosis. Dr. Brown has had good results from the injection of the hard lumps that form with tincture of iodine, using a strong hypodermic syringe to make the injection. Drs. Stewart, Miller, Titus, and others spoke on the subject.

On motion by Dr. Kingery, being duly seconded and carried, the election of officers was then taken up, the result being the election of Dr. H. Shipley, of Sheldon, Iowa, for President; Dr. H. E. Talbot, of Des Moines, Iowa, First Vice-President; Dr. P. O. Koto, of Forest City, Iowa, Second Vice-President; Dr. John E. Brown, of Oskaloosa, Iowa, Secretary and Treasurer. Board of Censors—Dr. Whitbeck, Decorah; Dr. S. K. Hazlet, Oelwein; Dr. H. L. Stewart, Oakley.

Dr. W. H. Austin, of Newton, Iowa, then read a paper on "Diseases of the Udder and Teats of Cows." Discussion followed. Some of the members present thought the enlargements in the milk-ducts were due to tubercular deposits, and were also the cause in many cases of stringy and bloody milk. Dr. Johnston has seen quite a number of such cases and made tuberculin tests on them, and has failed to get a reaction in any case. For such cases he gives Fowler's solution and potassium nitrate for eight days, then skips a few days and takes up the treatment again. When abscesses form in the udder he extirpates the section. Dr. Parslow thinks some of these enlargements in the teats are polypi.

Dr. S. Whitbeck being called to read his paper on "Nitro-glycerin: Its Actions and Uses," stated that he had not completed his experiments with the drug, and promised to produce a paper on the subject on some future occasion.

Dr. G. A. Scott, of Independence, Iowa, who was on the programme for a paper on the subject of "Filaria Oculi Equi," was unavoidably absent, but had forwarded his paper, which was read by the Secretary. Discussion followed regarding eye-operations—puncturing the cornea for treatment of specific ophthalmia, etc. Dr. Miller says there is little danger, and he frequently punctures the coats of the eye in treating this form of ophthalmia, with good results if performed in the early stages. If there have been several attacks the operation is useless. Dr. Peters has operated with complete success. The cornea is tough, and it takes a pretty sharp knife to go through.

Dr. R. R. Hammond, of Le Mars, Iowa, then read a paper on "Amputation of the Uterus in Cows." Discussion followed regarding similar cases, treatment, trusses, etc., Drs. Drinkwater, Austin, Stewart, Brown, and others taking part.

Dr. Peters, of Lincoln, Neb., chairman of Committee on Organization of the Trans-Mississippi Veterinary Association, reported correspondence on the subject. It seemed to be the opinion that such an organization could not be maintained without detracting from the interest and attendance of the American and State Associations. Talks were made by quite a number of the members, and the kindly feeling existing between the Nebraska and Iowa Associations frequently referred to, and all seemed to favor the holding of joint meetings on certain occasions. Dr. Shipley made a motion, which was seconded by Dr. Austin, to receive the report and continue the committee to arrange for a joint meeting of the associations of Nebraska and Iowa at some future time. Dr. Brown moved to amend by adding: "And give the committee power to call a joint meeting of the two associations when, in their opinion, an opportune time has arrived." The amendment was accepted, and the motion as amended was voted and passed. At the request of Dr. Peters the Secretary of the Iowa Association, Dr. Brown, was added to the committee.

The Association extended a vote of thanks to Dr. Talbot for the interest he had shown and the efforts he had made for the success of the clinical session.

Dr. Whitbeck, as chairman of the Committee on Resolutions, reported the following: 1st. *Whereas*, Questions of very great importance often arise at a time when the voice of this Association cannot be heard in convention assembled; therefore, be it *Resolved*, That the President is hereby instructed to appoint a committee of three, in addition to the President and Secretary, who shall be *ex-officio* members, this committee to be known as the Judiciary Committee, and shall have jurisdiction in all matters concerning this Association except at such times as the Society may be in session. 2d. *Whereas*, The trustees of the Iowa Agricultural College have increased the work of the professors in the veterinary department to the extent of requiring the teaching of the subjects of pathology, therapeutics, comparative anatomy, surgery (operative and general), practice of veterinary medicine, obstetrics, meat-inspection and milk-inspection, veterinary materia medica, examination for soundness, in addition to other veterinary work—laboratory and otherwise—of a well-equipped veterinary college; and *Whereas*, In addition to this they not only propose to continue but increase the experimental work of the station; therefore, be it *Resolved*, That it is the sense of the Iowa State Veterinary Medical Association that this amount of work is inconsistent with the quality of service that should be required, and if it be necessary to require this work of two men, then only such men as are fitted by years of training with the practical and theoretical phases of veterinary science should be employed. And be it further *Resolved*, That until so equipped it cannot continue in good standing with this Association. And be it still further *Resolved*, That we deplore the action taken by the trustees of the State Agricultural College in disposing of the services of one of the ablest men in the West in experimental and scientific veterinary medicine. By vote of the Association both resolutions were unanimously adopted.

Dr. Whitbeck moved the appointment by the chair of a committee of three to supply our national legislators with suitable literature bearing on army legislation, and that our members be asked to use their influence with their Congressmen; seconded and carried. On motion of Dr. Gibson, Dr. A. T. Peters was added to this committee.

Dr. Peters was given a vote of thanks for coming and for the interest he has shown in our Association.

A vote of thanks was also extended to the management of the Savery House for the room and for other courtesies during our sessions.

Moved by Dr. Whitbeck that a committee of three be appointed on publication of reports, and that the same be published if possible. This was seconded and carried.

President Shipley then announced the following committees:

Army Legislation: Dr. P. O. Koto, of Forest City; Dr. S. K. Hazlet, of Oelwein; Dr. A. S. Brodie, of Cedar Falls; Dr. A. T. Peters, of Lincoln, Neb.

Judicial Committee: President, H. Shipley, Secretary, J. E. Brown (*ex-officio*); Dr. J. I. Gibson, of Denison; Dr. C. E. Stewart, of Chariton; Dr. J. G. Parslow, of Shenandoah.

Sanitation Committee: Dr. W. B. Niles, of Ames; Dr. J. I. Gibson, of Denison; Dr. S. T. Miller, of Shelby.

Committee on Disease and Treatment: Drs. H. E. Talbot, of Des Moines, S. H. Kingery, of Creston; R. R. Hammond, of Le Mars.

Committee on Speakers for Farmers' Institutes: Dr. W. B. Niles, of Ames; Iowa.

There being no further business, the meeting adjourned to meet in Des Moines next fall or winter, at the call of the President and Secretary.

JOHN E. BROWN,
Secretary-Treasurer.

### CHICAGO VETERINARY SOCIETY.

THE regular monthly meeting was held January 12th. President Robertson presided, and the following members were present: Drs. Allen, Campbell, Clancy, Dubia, Fish, Howe, Hughes, McCoy, Merillat, McGarth, Paxson, Pierce, E. L. Quitman, Robertson, Ryan, Walker, Wingate, and Worms.

Following the reading and approval of the minutes of the previous meeting the Legislative Committee presented a report recommending all members to call upon the Representatives and Senators of their respective districts, and as individuals to exercise all possible influence for the advancement of a bill to be presented to the State Legislature for the regulation of the practice of veterinary medicine throughout the State. A motion was presented and carried for accepting the report, and the Secretary was ordered to notify all members not present of the committee's recommendation.

The resignation of Dr. Joseph D. Tuthill was read and accepted with regret.

The conclusion of Dr. L. A. Merillat's excellent paper on "Soundness" was received with enthusiastic applause, and a lengthy and spirited discussion followed, which was participated in by Drs. Hughes, Walker, Robertson, Ryan, Quitman, and many others.

Many cases were reported and discussed by several of those present.

The chairman of the Literary and Publication Committee announced that Dr. R. G. Walker would present a score or more subjects and their relation to soundness at the next monthly meeting.

There being no further business to enter upon at so late an hour, adjournment was ordered.

*Conclusion of Dr. Merillat's Paper on Soundness.*

Among the subjects assigned me are balkiness and side-pulling. These have already been discussed at length, and therefore require no reconsideration. The remaining topics for discussion to-night are roaring, whistling, snoring, grunting, heaves, cribbing, weaving, chorea, and paresis.

*Roaring* is a name used to denote the abnormal inspiratory sound manifested in horses during more or less severe exertions. It is the effect produced by a stenotic condition of some part of the upper air-passages, which may be classified according to location into nasal, pharyngeal, laryngeal, and tracheal.

*Nasal stenosis* is due to some pathological condition of the nasal cavities, sinuses, or their lining membrane.

*Pharyngeal stenosis* is usually the result of compression of the pharynx by abscesses or tumors in the guttural region.

*Laryngeal stenosis*, in 95 per cent. of all cases, is due to vapidity of the left arytenoid cartilage and vocal cord, but may be the result of polypi or any neo-formation.

*Tracheal stenosis* is ordinarily the result of a previous tracheotomy or of accidental traumatisms causing, by an inflammatory process, collapse of the tracheal rings. Hyperplastic conditions within the trachea are rare entities. Animals that have been subjected to tracheotomy are fit subjects for condemnation, as stenosis, especially in young animals, is a frequent sequel. Purchasers must at least be told of the possible result.

*Roaring* from any of the above or similar conditions must be considered an unsoundness, without reservation or exception.

The abnormal respiratory sounds occurring along the course of acute catarrhal affections, and which disappear with the morbid processes, are not to be dealt with too harshly, and animals so affected might justly be referred to future examination; but any abnormal sound following in the wake of such diseases, although they often gradually abort, must always be regarded with suspicion. The veterinarian is never justified in risking a prognosis in such cases.

*Whistling* is a synonym for roaring.

*Grunting* is evidently intended to denote the short expiratory sound observed in horses when executing a sharp turn. This sound points to pleural adhesions, the result of a previous pleuritis. It usually follows infectious pleuritis, and is frequently associated with roaring. In the horsedealers' vocabulary such animals are called "bulls." The grunting varies with the extent of the lesion, which in many cases renders horses almost useless for heavy or fast work, especially during the warm months. Prolonged panting after exertion is ordinarily produced by this condition. Horses so afflicted are unsound, but acute grunting occurring during the preliminary stages of pleuritis, pericarditis, pink-eye, purpura hemorrhagica, and rheumatism must be differentiated from the chronic.

*Snoring.* If snoring in the equine species is analogous to the ungodly guttural sound produced by the *genus homo* during his slumbers, then I must regard it as evidence of sound health rather than that of unsoundness. If there is any one thing that savors of good health more than another it is loud snoring. In this I refer to the human, and no doubt the same deduction will apply to the horse. In any event, at $2 *per capita* the examining veterinarian would hardly be expected to sit up nights and patiently wait for the horse to slumber in order to determine whether or not he is an habitual snorer.

*Sniffling,* although not mentioned on my list, deserves notice, because it is a condition which may be entirely overlooked in an examination. This sound is observed during rest, and may be entirely absent during exertion. It is usually the result of circumscribed hypertrophy of the nasal septum near the anterior nares.

Another condition closely allied to the foregoing is *atrophy of the nasal dilators,* due to paralysis of their motor nerve. The nostril, being unable to dilate, is drawn inward during each forcible inspiration, causing a dyspnœa so severe as to almost suffocate the animal. In partial paralysis these symptoms are modified in proportion to the degree of paralysis, and might be so slight as to be mistaken for less serious conditions.

*Heaves* is a dilatation of the pulmonary alveoli, resulting from a variety of functional or pathological derangements. It is clinically manifested by a double expiratory effort to expel the tidal air from the dilated cells. Heaves, however trivial, is a condition which must condemn any animal so afflicted. It can be controlled by careful management, but is never entirely cured; and with hard or fast work, careless dieting, or improper sanitation, will sooner or later render an animal useless.

*Cribbing* is a habit induced by idleness, and is of two kinds: First, cribbing by support; and, second, cribbing without support. The latter is also called *wind-sucking.* The former often terminates in the latter. Cribbing by support is executed in about the following manner: The head is fixed by resting the upper incisors upon some fixed object. Air mixed in the mouth with saliva is rapidly forced by suction into the pharynx, which has been dilated by forcible contraction of the omo-hyoideus and sterno-thyro-hyoideus muscles. As these muscles relax the larynx quickly assumes its normal position, and thus with the contraction of the pharynx forces the air into the œsophagus. Cribbing without support is the same act minus biting.

Cribbing is not an unsoundness, and if the habit did not occasionally result in cœliac diseases it might be entirely overlooked in examinations. Prejudice, however, forces the veterinarian to reject all cribbers.

*Weaving* is also a habit caused by idleness. It should not be considered sufficient reason for condemning a good horse. In this I will probably not be upheld by the majority of veterinarians, but I take this stand because weavers are usually high-class horses with unusual power of endurance.

*Chorea* is a disease of the corpora striata and optic thalami, often extending to the cervical cord. (This is the statement of recent writers.) Its clinical manifestation is that of muscular delirium. It is a common affection of man and the dog, but is rare in the horse. Being a disease with a chronic course, its possessors must be stamped as unsound.

*Paresis.* The general diminution of nerve-force originating in the higher nerve-centres, and exerting its effect not only upon the psychic but also the motor and sensory functions of the whole body, is a condition I have never observed nor heard of in the horse. Chronic hydrocephalus resulting from an exudation or transudation into the lateral ventricles might be said to cause a paresis, but I fear this nomenclature will not bear up under critical discussion. If, however, the word is intended to denote a partial peripheric nerve paralysis, para-paresis, or tabes dorsalis, then I can dismiss the subject by simply stating that all such diseases I determine a decided unsoundness.

*Discussion.*

Dr. Hughes: I congratulate Dr. Merillat on having so thoroughly covered the ground, and am sorry that he has not given us a little more opportunity to criticise his effort. At the same time there are a few points in which I do not exactly agree with him. Regarding snoring, for instance, Dr. Merillat expresses himself rather lightly. It is a very disagreeable habit, and one which markedly lessens an animal's value, and we do not have to stay up all night to hear an animal snore. Snoring in a horse is due to vibration of the membrane between the false nostril and nasal chamber proper. That it may be acquired I have demonstrated to myself by having seen one horse contracting the habit from another, owing to standing in an adjoining stall. The horse referred to snored while standing quietly in the stable, but rarely did so on the street. Dealers are always afraid that a snoring horse will be rejected, and when trotting them out to exhibit them to an intending purchaser will rub their hands down over their nostrils as soon as they bring the horse to a standstill, thus stopping the tendency to snore or snort. Dealers, too, having a pronounced snorer or snorter put compresses, attached to the nose-band of the bridle, over the false nostrils, and in this manner prevent it.

In regard to grunting, I cannot subscribe to the theory that it is always caused by thoracic adhesions. I have held post mortems on some pronounced grunters, and in two cases have found it was produced by cirrhosis of the liver, one of these cases having a liver weighing nearly forty pounds. There were no pleuritic adhesions in either of these cases, nor were there any abnormal lesions of the larynx present.

Regarding cribbing, I would like to ask whether it is possible for a horse to swallow air. According to our essayist, it is. Is it possible for the pharynx to contract a volume of air so as to force it down the œsophagus, considering that such air has so many ways of escape? What we do know is that some cribbers fill up with air, and whether it is liberated as a consequence of indigestion, or whether the air is sucked down the œsophagus, is a question I have been unable to solve.

Regarding chorea, I was surprised to hear the essayist make the statement that it is rare in the horse. I frequently meet with it in my practice. If chorea is rare in the horse, then I have for years been regarding wrongfully a certain condition as chorea. The condition I refer to is that peculiar spasmodic twitching and contraction of the muscles of the hind limbs or tail, exhibited when a horse is backed up or turned around, or sometimes seen when the animal is standing quietly in the stall.

Dr. Merillat: As to snoring I agree with Dr. Hughes, except in the nomenclature of the condition. The term snoring, described by all physiologists, is a guttural sound produced during slumber; consequently I took it for granted that you wanted a description of that condition. The trouble Dr. Hughes described is due to muscular atrophy, which is a very common condition. I have a case in mind that was so aggravated that it nearly suffocated during exertion. I do not call this condition snoring. I took snoring to mean nothing else but snoring. If you can get a name for a noise between sniffling and snoring you can name the condition Dr. Hughes describes.

Regarding grunting, I have stated that it is usually due to pleuritic adhesion following infectious pleurisy, and affecting the left inferior laryngeal nerve, which accounts for its frequent association with roaring. Grunting may also be caused by abdominal lesions. One grunter I held a post mortem on was found to have an adhesion between the colon and parietal peritoneum. That animal was a fearful grunter. In my experience, however, I have found that a pleuritic condition is the more frequent cause of grunting.

In writing these remarks I was at sea in dealing with the word paresis. Paresis as a general disease or a primary condition is, I believe, the name applied to a form of human insanity not recognized in the lower animals. The word paresis, of course, might be used to express partial paralysis of special nerves, all of which, as stated in my paper, must be regarded as serious conditions.

As to swallowing air, I am in the same fix as Dr. Hughes, as far as my own observations are concerned. I have, however, observed that when a bloated cribber is tapped with a trocar and canula the gas will not ignite, and has not the characteristic smell of the usual intestinal gases.

Dr. Quitman: I want to add my evidence to this last statement. First, there is no doubt that a human being can swallow sufficient air to swell himself up. In fact, I know a man on the West Side who does it for fun. If it is possible for a man it ought to be for a horse. The gas seems to be much cooler than that of fermentation. Then the horse does not show the intense distress that the gas of fermentation would cause.

Paresis is a word that is used in a very broad sense. If I am not mistaken it was originally meant to describe a cerebral softening, but now commonly used to describe a local affection. In regard to cribbing, inasmuch as it happens so often that it turns into windsucking, I think it ought to be called an unsoundness.

Locomotor ataxia is sometimes found in horses. One horse that I have recently condemned was probably the most beautiful case that I ever saw. As far as I could trace it the case was in existence for about three years, and he was useless for over a year. He lacked coördination of all four limbs, and if blindfolded and backed he would collapse in a heap. In fact, I have seen a number of similar cases that I would term locomotor ataxia.

Dr. Merillat: Oh, no! Chorea can be cured with time. In man, with age it entirely disappears.

Dr. Campbell: Have you ever seen a case of St. Vitus' dance in a horse?

Dr. Merillat: I think I have.

Dr. Hughes: As to windsucking, those who are in the habit of tapping horses have noticed that it is a very common thing not to be able to light the gas. This is found also in horses that are not cribbers.

In regard to chorea, Dr. Merillat refers to it as the same disease found in man. He mentions, though, that chorea of the dog is entirely different in its symptoms from that of man. Why not go a little further and call this crampy condition so often met with in horses also chorea. We should endeavor, not so much from a pathological as from a practical stand-point, to find a suitable name for these different conditions. As in chorea, a decidedly crampy horse gets worse right along; hence it is a progressive disease, and it will assume an acute form sooner or later if, for instance, such an animal be attacked with pneumonia or any acute disease. The complication always lessens the tendency to recover, and often proves fatal. I always associate rheumatism with chorea, and find it a very difficult matter in many cases to differentiate them. From a practical stand-point I would ask Dr. Merillat to give a technical name for the condition of a horse that is commonly called "crampy." I examined a horse recently for soundness at the stock-yards, and in my certificate stated that the horse had chorea. The case was a very pronounced one, and I naturally rejected him. I was asked by the seller if chorea meant crampy, and I said yes, as that is the name under which the disease is known to ordinary horsemen. What technical term would Dr. Merillat use to designate such a condition?

Dr. Merillat: I would call it stringhalt.

Dr. Hughes: In stringhalt there is a clean, forward automatic movement, with exaggerated flexion. Where there is a hindward muscular action, the animal swinging his legs spasmodically outward, I would regard it as chorea. The first condition is generally regarded as a local, nervous one; the second as a disease in the great nerve-centre.

Dr. Merillat: If you wish to call all these little crampy conditions chorea I have no objection. Chorea is a disease of a chronic course, and we have no right to call these crampy conditions chorea, although I know that many authors class them as chorea, though they never fail to state that it is also stringhalt.

Dr. Ryan: As to locomotor ataxia, we often get conditions like that from digestive trouble. I think Dr. Hughes's suggestion, to apply the term chorea to conditions as described above, is a good one. We can safely use it. To say a horse has stringhalt or shivers would not be correct. We cannot expect a horse or dog to have a typical case of St. Vitus' dance, and if the cases are similar there should be no objection to their being called chorea.

Dr. Hughes: I would like it if we would come to a decision in regard to this matter. Suppose I examine a horse and state that he has chorea, while another veterinarian would call it a crampy horse. This would cause a considerable amount of damage to a man's reputation. Chorea, as a rule, gets worse with age; hence every horse showing even the slightest symptoms of it should be condemned.

Dr. Ryan: Is it not a poor way, in cases of grunters caused by cirrhosis of the liver or enlargement of the heart, to certify it being a grunter? Why not specify the cause?

Dr. Merillat: It would be a happy state of conditions if we could.

Dr. Quitman: In regard to chorea of man and dog, I believe them to be identical. You will find in a dog affected with it that his foot will go first one way and then another. It is the same way with the human sufferer if, for instance, he tries to put a spoon in his mouth. The similar conditions in the horse I would call "choreaic affections," which would imply that it resembles chorea. I had a case of a horse that had a cramp in one hind leg. I used all methods imaginable—used the whip, etc., but could not break the cramp. It existed ten days in one leg, then it jumped into the other. What would you call that? I advised the owner to get rid of the horse.

Dr. McCoy: I think Dr. Quitman's client evidently got rid of him, for I saw a similar case on the street the other day. As to cribbing and weaving, are they diseases that come entirely from idleness?

Dr. Quitman: I am inclined to believe that cribbing of young horses is sometimes brought on by irritation of the teeth. It seems that they will attempt to draw the air in to cool the inflamed gums. We know that older horses sometimes take it, but it is evidently a habit.

Dr. Merillat: Friedberger and Fröhner's *Pathology and Therapeutics* gives three causes—heredity, idleness, and association. They also quote a large percentage of army horses that became cribbers from idleness.

Dr. Clancy: In regard to a proper name for this crampy condition, why not call it pseudo-chorea?

Dr. Hughes: Pseudo is a prefix that in my opinion should not be used. Call it chorea, and settle every doubt.

JOSEPH B. CLANCY,
Secretary.

### KEYSTONE VETERINARY MEDICAL ASSOCIATION.

THE March, 1898, meeting was held in connection with the annual meeting of the Pennsylvania State Veterinary Association, on the 8th instant, under what was undoubtedly the most pleasant auspices, with the largest attendance that has ever graced a meeting of this Association. Its large attendance was due to the fact of its being called to order by President Leonard Pearson, who was also host at the largest banquet-table ever surrounded by veterinarians, there being over one hundred and twenty assembled to testify to the popularity of the host and to emphasize the fact that they appreciated a gastronomic treat far more than a literary one. Secretary Rhoads promptly moved to adjourn, which was carried, and thus began and ended the briefest, best-attended, and undoubtedly the most popular meeting of the Keystone Veterinary Medical Association.

THE April meeting of this Association was called to order by President Dr. Leonard Pearson, with the following members of the profession present:

Drs. Otto Von Lang, Grubb, W. E. Mecray, S. J. J. Harger, H. D. Hackler, McCoy, Stauffer, Vasey, John B. Raynor, Felton, W. H. Hoskins, Leonard Pearson, W. H. Ridge, Charles Lintz, Charles T. Goentner, J. W. Adams, H. P. Eves, James B. and Thomas B. Rayner, C. J. Marshall, J. D. Houldsworth, and J. Cheston Morris, M.D.

Secretary Rhoads being absent, on account of sickness, the reading of the minutes was dispensed with, and the Secretary's duties were performed by Dr. Ridge.

After the general routine of business the March meeting of the Keystone and State Association and the banquet given by Dr. Pearson were discussed.

Dr. Pearson gave a talk on the veterinarians of foreign armies, giving their rank and standing from the time of the Roman army to the present.

Dr. James B. Rayner then read a report of a case of "Paralysis of the Bowels." After the discussion on this paper the meeting adjourned to meet on May 10, 1898.

W. H. Ridge,
Secretary pro tem.

The May, 1898, meeting of this Association was called to order by President Dr. Leonard Pearson on the 10th instant, at 8 P.M., with the following members of the profession present: Drs. James M. Mecray, S. J. J. Harger, H. D. Hackler, Otto von Lang, Vasey, John B. Raynor, H. B. Felton, M. W. Drake, Charles T. Goentner, W. L. Hart, W. H. Hoskins, Charles Lintz, L. Pearson, W. L. Rhoads, Thomas B. Rayner, William Ridge, C. J. Marshall, John W. Adams, A. N. Lushington, J. D. Houldsworth, and J. Cheston Morris, M.D.

After the general routine of business Dr. W. H. Hoskins gave a most interesting talk on "Emphysema," covering his subject so well that Dr. Pearson could scarcely get a word from any of the members or visitors on the subject, yet the discussion brought out diverse opinions as to lesions and treatment.

The subject of the army veterinarian again came up for discussion. Dr. Hoskins moved that the officers of the Keystone Veterinary Medical Association confer with officers of the State Association as to the best method of raising $200 from the profession throughout the State for furtherance of army legislation. The President appointed Drs. Hoskins and Rhoads as a committee of two to take this matter in charge. They made an appeal to those present, and raised $30. Dr. Rhoads moved that the President appoint a committee of three to draft resolutions directed to Dr. Salmon, pledging the support, morally and financially, of the Keystone Veterinary Medical Association in this work.

The application for membership of Dr. James M. Mecray, of Maple Shade, N. J., having been favorably reported by the Board of Censors, was now voted upon, and he was unanimously elected to active membership.

The Association now adjourned to meet on June 14, 1898, at which time Dr. Harger will open a discussion on "Quittor."

The June, 1898, meeting of this Association was called to order by President Dr. Leonard Pearson on the 14th instant, at 8 P.M., with the following members of the profession present: Drs. Charles T. Goentner, W. H. Hoskins, W. G. Shaw, T. B. Rayner, S. J. J. Harger, Charles Lintz, J. W. Adams, Nicholson, J. M. Mecray, McGary, H. D. Hackler, Land, Cunningham, Spaeth, P. K. Jones, Spindler, M. W. Drake, W. H. Ridge, and W. L. Rhoads.

Dr. John W. Adams read a paper on "Operative Treatment for Cribbing," and stood the test of all sorts of questions from all points of view bringing out many salient points and valuable suggestions.

Dr. S. J. J. Harger opened the discussion on "Quittor." This subject was well entered into, thoroughly sifted, and discussed.

Dr. W. H. Hoskins and others thoroughly aired the recognition the army veterinarian should receive but does not get.

The meeting adjourned to meet September 16th, as this date would not conflict with the trip of those members who attend the Omaha meeting, at which time Dr. Leonard Pearson will give a talk on "Veterinary Notes from Europe."

The September, 1898, meeting was held on the 16th instant, having been postponed from the 13th in order that our members who attend the Omaha convention might have time to return and tell of the sights and pleasures; but, sad to relate, their guide lost his bearings going out, and travelled hundred of miles around. Those who arrived on time became so engrossed with their initiation into one of the large Western lodges and their progression in the art of camel riding that the Keystone Veterinary Medical Association meeting was as yet a week too soon for them. Our President being absent, Dr. J. T. McAnulty was called to the chair, and the meeting convened with the following members of the profession present: Drs. T. B. Rayner, James B. Rayner, John B. Raynor, Otto Von Lang, Joseph D. Houldsworth, S. J. Nicholson, H. B. Felton, J. T. McAnulty, John J. Repp, J. M. Mecray, and W. L. Rhoads.

After the general routine of business the Secretary asked for information regarding the start and early history of the Keystone Veterinary Medical Association, for use in the By-Laws, etc., of the Association about to be printed.

Dr. James B. Rayner reported a case. Dr. J. T. McAnulty reported a case of "Lameness," which had been nerved and afterward fired, and seemed to be doing well. Dr. Houldsworth also reported a case of lameness.

Dr. Mecray reported a case of "Staggers." Post-mortem examination revealed a red tumor, the size of a pea, attached to the dura mater and pressing upon the brain. The diversity of reports and discussion thereon brought up points far from the original topic, yet at all times adding new interest and making this one of the most pleasant meetings held for some time.

Meeting adjourned to meet October 11, 1898.

The annual meeting of 1898 was called to order by President Dr. Leonard Pearson, at 8 P.M. on October 11th, with the following members of the profession present: Drs. E. S Muir, W. H. Hoskins, H. P. Eves, S. J. J. Harger, W. H. Ridge, C. J. Marshall, C. T. Goentner, E. M. Ranck, J. M. Mecray, W. G. Shaw, J. D. Houldsworth, J. W. Adams, Leonard Pearson, R. A. Hummel, and W. L. Rhoads; also Mr. Pearson.

After the roll-call the reading of the minutes was dispensed with. Dr. John J. Repp was elected to active membership and introduced to those present.

It was moved by Dr. Hoskins, and carried, that the incoming President appoint a legislative committee of five, with recommendations that they pay particular attention to any legislation regarding the practice of veterinary medicine in the State, also questions on meat-inspection and milk-inspection.

In response to the Secretary's appeal for information regarding the early history, etc., of the Keystone Veterinary Medical Association it was voted that the incoming President appoint a committee of three original members to procure the original minute-book, if possible; if not to make a report from memory of the origin of the Association, said report to be embodied in the reprint of the Constitution and By-Laws, etc.

The President asked for a report from the committee appointed at the January meeting to ascertain the truth of the statement on milkmen's advertisements that dairies were under veterinary sanitary supervision. After their report they were instructed to give a written report at the January meeting.

The Treasurer being absent, no report was made. The Secretary's report showed a balance of $10.69 in his hands after the payment of all bills to date.

Dr. Ridge was now called upon to read his paper on "Œosphagotomy." This paper was received with much interest, and the discussion brought forth many means of treatment before performing the operation, among which the employment of veratrum and pilocarpine was prominent.

Dr. E. S. Muir now read a paper on "Hypodermic Cathartics,"[1] which was based upon a series of experiments made at the veterinary department of the University under his personal supervision. It was made not alone as a clinical illustration, but more for a further advancement in the art and science of the use of hypodermic cathartics. The paper concludes as follows: "We find on consulting the table that barium chloride acts quickly (from one to nine minutes), causes large quantities of feces to be voided, does not cause pain, and the action ceases in from one to three hours. Physostigmine sulphate causes abdominal pain, is longer producing evacuation, its action lasts from two to four hours, causes expulsion of large quantities of feces, mucus, and flatus; while arecoline acts quickly, it causes more pain than either of the other salts, action lasts longer (from three to five hours); amount of feces voided small, never very soft; but it produces profuse flow of saliva and an involuntary dripping of urine." After considerable discussion on this valuable paper the Association took up the election of officers for the ensuing year, with the following results: President, Dr. Leonard Pearson; Vice-President, Dr. H. P. Eves; Secretary, Dr. W. L. Rhoads; Treasurer, Dr. Charles T. Goentner. Censors: Drs. W. H. Hoskins, C. J. Marshall, J. W. Adams, W. H. Ridge, and Thomas B. Rayner. After the election of officers the Association adjourned to meet November 8, 1898.

THE November, 1898, meeting was called to order on the 8th instant by President Dr. Leonard Pearson, with the following members of the profession present: Drs. H. P. Eves, C. Williams, S. J. J. Harger, J. D. Houldsworth, C. J. Marshall, E. M. Ranck, W. H. Hoskins, Leonard Pearson, E. H. Landis, W. G. Shaw, J. W. Adams, W. L. Rhoads; also Mr. R. A. Pearson.

[1] See page 26, January, 1899, and page 87, February, 1899.

President Pearson announced his committees for the year, as follows: Committee on Programme—Drs. W. L. Rhoads, J. J. Repp, and J. W. Adams. Committee on History—Drs. C. T. Goentner, W. H. Hoskins, and W. B. E. Miller. Committee on Legislation—Drs. W. H. Hoskins, F. S. Allen, W. S. Kooker, James B. Rayner, and W. H. Ridge. Committee on Army Legislation—Drs. C. J. Marshall, Francis Bridge, and H. P. Eves. Investigation Committee (continued)—Drs. C. J. Marshall, J. D. Houldsworth, and Charles Lintz.

After the regular routine of business Dr. E. H. Landis gave some interesting reminiscences of army life during the late war, showing the standing of the army veterinarians and the reasons therefor. Their appointment is due invariably to political influence, and after being appointed the graduate has no opportunities to prove that qualified men are superior to bricklayers or mule-drivers, as they have no facilities, no drugs or medicines, and worse than no authority. He told to what a great extent nitrate of potassium was used and with what dire effect, and also spoke of the elaborate and far from efficient mallein-test made; glanders and distemper were diagnosticated as the same, to simplify treatment. He gave some examples of reports made to heads of departments. A vote of thanks was extended to Dr. Landis, and it was moved and seconded that we, as an association, do all that is possible to improve the condition of affairs.

Dr. Pearson now spoke on the standing of army veterinarians in foreign countries and the steps through which they came. He gave the French the credit of having the best-mounted cavalry in the world. He then gave a talk on "Gems and Germs from Germany." He spoke of the great activity there in veterinary work, of the complete system under which they work, free from political influence. He also spoke of our inadequate meat-inspection and milk-inspection, and cited the loss of 100,000 hogs in Cumberland County from hog-cholera, and of the losses to our local cattle-feeders through inadequate means of stopping Texas fever. His talk was instructive and full of humor.

The Investigating Committee now made a report, and Dr. Charles Williams gave a brief résumé of "Veterinary and Sanitary Inspection of Milk in Philadelphia."

Mr. R. A. Pearson spoke of "Laws and Ordinances Governing Inspection of Milk in Different Municipalities."

The Board of Censors having favorably passed upon the application of Dr. W. G. Shaw for active membership, it was now voted upon by the Association, and he was unanimously elected to membership.

The meeting adjourned to meet December 13, 1898.

THE December, 1898, meeting was called to order by Dr. W. S. Kooker, Dr. Leonard Pearson and Vice-President Dr. Eves being absent. Those who were there at roll-call or came in later were: Drs. Francis Bridge, H. P. Eves, C. Williams, J. M. Mecray, Otto Van Lang, J. J. Repp, W. G. Shaw, George Felton, John B. Raynor, J. T. McAnulty, J. W. Adams, E. M. Ranck, S. J. J. Harger, Otto G. Noack, W. H. Ridge, W. L. Rhoads, H. D. Hackler, W. H. Hoskins, W. H. Super, and C. J. Marshall. After the general routine of business the subject for discussion, "Founder," was taken up by Dr. Charles Williams, who held that a horse must first have some systematic or constitutional disturbance, and founder was but a sequel to these, and not a primary trouble. He was followed by Dr. Bridge, and the discussion was entered into with zest by everyone, and the causes, conditions, cures, etc., were varied and well worth being considered. The causes were given as overdriving, chilling by draft, water, or feeding, etc.; others cited cases of mares running in fields with foal, and others after abortion and parturition. It was divided into two forms—chilling and rheumatoid. This theory found its advocates, and there were others claiming that, though there were many causes, there was but one founder. It was claimed to be often confounded with tenosynovitis and myositis. The treatment varied. There were advocates of purging and those who were against it. Remedies were from large and repeated doses of nitrate of potassium, aconite, aloes, calomel, fleam, salol, pilocarpin, salicylic acid, acetanilid, and iodide of potassium. Some advocated hot water, and others use ice-water.

The question of "Horn-growth" was considered, and the best methods of softening and growing hoofs. Dr. Hoskins spoke of the operation done by Dr. Lyford at the national meeting. Dr. Adams, having spent some time with Dr. Lyford, fully explained the operation, but did not think it anything wonderful or new.

Dr. Harger exhibited an interesting specimen of "Impaction of the Descending Portion of the Large Colon of a Newfoundland Dog," about one foot long by four inches through. The contents were pulpy and fibrous. The dog had suffered with constipation for two years, but had a good appetite until within two days of his death.

Dr. Ridge reported a case of "Abdominal Abscess in a Weanling Colt." There was some discussion as to the best way to improve meat-inspection and milk-inspection in Philadelphia; also the rank and standing of army veterinarians. It was moved and seconded that a press committee of three, also a committee of three to look after the heads of departments (official), be appointed for these and other matters which might crop up. Dr. Kooker, President *pro tem*, made the following appointments: Press Committee—Drs. Pearson, Hoskins, and Rhoads. Official Committee—Drs. Adams, Marshall, and Bridge.

The meeting adjourned to meet January 10, 1899.

THE January, 1899, meeting of this Association was callled to order by President Pearson, on the 10th instant at 8.30, with the following members of the profession present: Drs. S. J. J. Harger, Charles Williams, William H. Ridge, W.G. Shaw, B. M. Underhill, H. D. Hackler, Leonard Pearson, M. W. Drake, E. M. Ranck, Joseph D. Houldsworth, C. J. Marshall, W. L. Rhoads, S. McClure, W. H. Hoskins.

After the roll-call and reading of the minutes of the previous meeting Dr. Hoskins, as chairman of the Committee on Legislation, made a report urging all to make a concerted effort to assist in the movement to gain rank for the army veterinarian. Further plans were discussed for raising from the profession in Pennsylvania the two hundred dollars pledged by the Keystone to aid in this work.

The annual meeting of the State Veterinary Medical Association was talked over and a committee of three (W. L. Rhoads, J. D. Houldsworth, W. G. Shaw) appointed to represent the Keystone Veterinary Medical Association in the entertainment of the State Association.

Dr. J. J. Repp having left the city, Dr. C. J. Marshall was appointed to act on the Programme Committee.

Dr. S. J. J. Harger was introduced and gave a very interesting talk on "Cribbing: Its Causes and Cures." He spoke of the numerous mechanical methods for preventing this vice, and fully described the operative treatment for its alleviation or cure, viz., the resection of the eleventh pair of cranial nerves or the resection of the sternohyoid and sternothyroid muscles. He reported four cases operated upon: first, by resection of the two muscles in front of the neck. The case improved, but was not cured. Second, roan gelding, same operation; improved, but not cured. Third and fourth, resection of the muscles and spinal accessory nerves. This proved a cure in each case up to the present time; while operation has not been done very long, he could not say what the final result will be.

A vote of thanks was extended to Dr. Harger for his very able and instructive talk. Dr. Pearson reported three cases which had been operated upon in Professor Dieckerhoff's clinic of Berlin by the resection of muscles only. He says the double operation is original with Dr. Harger.

Dr. Pearson now reported a series of cases in the southern portion of the State which caused the death of 150 to 250 cattle on sixty or seventy farms. They died in convulsions in from four to twelve hours after first noticed. A study of the trouble led them to diagnose it as cornstalk disease. He then gave a history of the cornstalk disease so far as known in this country. It is said to kill ten to twenty thousand in Kansas every year.

Drs. Rhoads and Ridge each reported cases very similar to those reported by Dr. Pearson.

Dr. Ridge continued his report on abdominal abscess in the colt.

Dr. Hoskins told why it was of interest to the individual taxpayer to have the army veterinarian a commissioned officer.

Dr. Pearson appointed Drs. Hoskins, C. J. Marshall, and J. D. Houldsworth a committee to draft resolutions of sympathy and condolence, and forward the same to Dr. Thomas B. Rayner.

Dr. Houldsworth spoke of the great good done by our State Veterinarian, and moved that a committee of three be selected to act for the Keystone Veterinary Medical Association in any movement toward having him reappointed for another term.

He then selected Drs. Hoskins, Marshall, and Adams as that committee.

It was then suggested that Dr. Houldsworth also consider himself one of said committee.

The meeting now adjourned to meet February 14th.

The February meeting of the Keystone Veterinary Medical Association was not held, as the profession, with the rest of humanity, was snow-bound by the blizzard.

W. L. Rhoads,
Secretary.

### ILLINOIS VETERINARY MEDICAL AND SURGICAL ASSOCIATION.

The tenth annual meeting was held at the office of S. H. Swain, Decatur, Ill., January 12th and 13th. The meeting was called to order with the President, J. Osborne, in the chair. The following members responded to roll-call: Drs. S. H. Swain, J. Osborne, A. J. Bagley, F. Glassbrenner, J. B. Walsh, J. Conover, C. A. Hurlbutt, V. G. Hunt, A. Travis, I. M. Luzader, and C. E. Conover. Visitors present: C. P. Lovejoy, State Veterinarian, of Princeton; F. Bromley, of Sullivan; D. Seitz, of Bement. The minutes of the last meeting were read by the Secretary, and it was moved and seconded that the report be accepted; motion carried. The President delivered his annual address, touching upon many points and encouraging the members present. Four applications for membership were presented by the Secretary. After a brief examination the Board of Examiners handed in their report, two applicants being favorably recommended, which resulted in the election to membership of Drs. I. M. Luzader and C. E. Conover. The other two applicants, not being qualified to pass the examination, were rejected.

The election of officers and appointment of standing committees for the coming year were announced, as follows: J. Osborne, Nokomis, President; V. G. Hunt, Arcola, First Vice-President; J. B. Walsh, Monticello, Second Vice-President; A. Travis, Litchfield, Secretary; C. A. Hurlbutt, Stonington, Treasurer. Committee on Membership: V. G. Hunt, S. H. Swain, I. M. Luzader, S. D. Brown, and F. Glassbrenner. Committee on Programme: J. Osborne, A. Travis, and C. A. Hurlbutt. Committee on Arrangements: J. B. Walsh, S. H. Swain, and C. E. Conover. Committee on Legislation: J. Osborne, S. H. Swain, and V. G. Hunt.

Papers were presented by J. H. Wattles, of the Western Veterinary College, on "Amputation of the Tail;" and C. Osborne, student at the Ontario Veterinary College, on "Cerebro-spinal Meningitis."

The Society then adjourned until 8 A.M., January 13th.

*January 13th.*

Meeting called to order at 8 A.M., with President Osborne in the chair.

Dr. F. Glassbrenner read an interesting paper on "Gastritis," followed by S. H. Swain on "Bovine Rumenotomy," which was fully discussed by the Association.

Under the head of unfinished business, Dr. S. H. Swain, acting as Treasurer in place of W. A. Swain, read the Treasurer's report. Motion was made and seconded to accept the same; motion carried.

It was decided to hold the tenth semi-annual meeting at the office of S. H. Swain, Decatur, on August 2 and 3, 1899, after which the Society adjourned.

A. TRAVIS, V.S.,
Secretary.

### MAINE VETERINARY MEDICAL ASSOCIATION.

THE regular annual meeting was held at Hotel North, Augusta, Wednesday, February 11th, at 7.30 P.M., President West in the chair. Those present were Drs. West, Freeman, Joly, Huntington, Purcell, Russell, Stevens, and Salley. The minutes of the previous meeting were read and accepted.

Our veterinary registration bill was taken up by sections, corrected, and made ready for presentation to the Legislature.

Election of officers resulted as follows: President, Dr. W. L. West, of Belfast; Vice-President, Dr. F. L. Stevens, of Farmington; Secretary, Dr.

I. L. Salley, of Skowhegan; Treasurer, Dr. A. Joly, of Waterville; Executive Committee, Drs. Huntington, Purcell, and Freeman.

Dr. Freeman read a paper on "A Veteriarian's Duty to his Associates," which was a very good exhortation to each member to do his duty not only to the animals under his care, but to his associates both in and out of the meeting.

On motion by Dr. Joly, seconded by Dr. Purcell, to meet at Augusta on the date of the hearing of our veterinary bill by the Judiciary Committee, which will be February 14th, at 2.30 P.M., voted to adjourn to meet at Augusta in April.

I. L. SALLEY, D.V.S.,
Secretary.

### MONTREAL VETERINARY MEDICAL ASSOCIATION.

THE regular meeting of the Society was held January 26th in the library of the College, Dr. Charles McEachran occupying the chair. Drs. Alloway, McCarrey, Mullins, and Sugden were also present.

Mr. Kato reported some interesting cases of the "Broncho-pneumonic Form of Distemper" which had come under his notice during the past summer. These had all been treated, and made good recoveries.

After these had been discussed the chairman called upon Mr. Henderson, who presented a paper on "Charlatanism," which excited a very animated discussion, it finally being decided to bring the matter before the coming alumni meeting next month.

There being no further business, the meeting then adjourned.

A regular meeting was held on February 9, 1899, Dr. Baker presiding.

Mr. Gellatty reported a very interesting case of "Flatulent Colic," which he had treated with good success. This was followed by an essay, by Mr. McGregor, on "Actinomycosis." These subjects excited a very interesting discussion, followed by an address by the chairman, after which the meeting adjourned.

JAMES MCGREGOR,
Secretary-Treasurer.

### VETERINARY MEDICAL SOCIETY OF THE UNIVERSITY OF PENNSYLVANIA.

MEETING was called to order February 3, 1899, at 8.10 P.M. Mr. Taylor was appointed critic.

The meeting was addressed by Dr. J. W. Harshberger, the subject being "Aspects of Spanish Bull-fights and Cock-fights." This lecture was extremely interesting; he also illustrated his talk by photographs collected during one of his trips to Mexico. A vote of thanks was extended Dr. Harshberger.

Meeting adjourned at 9.30 P.M.

Meeting was called to order February 17, 1899, at 8 P.M. Mr. Hughes was appointed critic.

Literary programme of the evening consisted of a paper on "The Analysis of Milk," by Mr. Jacobs. This paper was highly appreciated by the members.

Next in order was the debate, the subject being, *Resolved*, That the Vaccine Treatment for Anthrax should be Abolished. Affirmative: Mr. Newcomer, '99; Mr. Horner, '00; Mr. Bassler, '01. Negative: Mr. Taylor, '99; Mr. Phillips, '00; Mr. Carlisle, '01. The judges were Messrs. Miller, Young, and Shore. They decided in favor of the affirmative. The Society as a body decided in favor of the negative. The general discussion was very good.

Meeting adjourned at 9.30 P.M.

L. A. NOLAN,
Secretary.

### MICHIGAN STATE VETERINARY MEDICAL ASSOCIATION.

THE seventeenth annual meeting was held at New Grand Hotel, Lansing, on February 8th. The President, Dr. Judson Black, of Richmond, delivered an excellent address. Professor D. King Smith, of the Ontario Veterinary College, gave an interesting talk on "Topics Relating to the Practice of Veterinary Medicine and Surgery, both in American and European Countries." Dr. A. Campbell, of Berlin, Ontario, described ordinances governing meat-inspection and milk-inspection in different cities. Professor Marshall, of the State Agricultural College, exhibited various bacilli of new and deadly contagious diseases under the microscope, while Dr. Dunphy, Michigan State Veterinarian, showed interesting specimens of tuberculous glands removed from cattle.

Papers were read by Dr. H. F. Palmer on "Meat-inspection and Milk-inspection," and by Dr. W. W. Thorburn, on "Electricity as Applied to Animals." Other papers and communications by Drs. Black, Hawkins, and others were followed by lengthy discussions.

The meeting was one of the best in the history of the Association, and a large number of new members were added. A committee was appointed to complete arrangements for a circulating library of the latest veterinary literature, and energetic steps were taken to procure legislation that will elevate the standard of the profession.

The following officers were elected: President, William Jopling, Owosso; Vice-Presidents, J. J. Joy, Detroit; P. G. Duff, St. Louis; William Burdick, Chesaning; Secretary-Treasurer, W. A. Giffen, Detroit, after which the meeting adjourned.

W. A. GIFFEN,
Secretary-Treasurer.

### VETERINARY MEDICAL ASSOCIATION OF NEW YORK COUNTY.

THE regular monthly meeting of this Association was called to order by President Robertson at 8.30 P.M., February 1, 1899. The following members responded to roll-call: Drs. Ackerman, Bell, J. S. Cattanach, Jr., Clayton, Delaney, Dickson, Ellis, Farley, Gill, Grenside, Hanson, MacKellar, O'Shea, Robertson, and Ryder. The minutes of the previous meeting were read and approved.

A quorum of the Board of Censors not being present, their meeting was deferred until after the reading of the first paper, which was presented by Dr. Ryder, entitled "Some Fine Points of Opinion in the Examination of Horses for Soundness." This paper was freely discussed by the members present, most of them agreeing with the excellent points brought out in the essay. Moved and seconded that a vote of thanks be extended to Dr. Ryder; carried.

Moved and seconded, that fifteen minutes' recess be granted for the Board of Censors to transact the details in hand; carried. So ordered by President Robertson.

Dr. Clayton, chairman of the Board of Censors, reported that in the case of Dr. Lamkin the Board sustained the findings of the previous Board at the December meeting, and recommended that it take the usual course, according to the By-Laws of the Association, and that the Board recommended the acceptance of Dr. J. S. Cattanach's resignation. Moved and seconded, that the report of the Board of Censors be accepted; carried Moved and seconded, that the Secretary notify Drs. Lamkin and Cattanach of the action taken; carried.

Dr. Ackerman then read a very interesting paper, entitled "The Hygienic Marketing of Milk," describing a method entirely new to the members, and exhibited a sample of milk bottled by this new process, which was afterward sampled by the members, and proved to be of a most excellent quality. After some discussion, which had to be limited for want of time, a vote of thanks was tendered to Dr. Ackerman.

Dr. O'Shea, chairman of the Judiciary Committee, then submitted the report from that committee notifying the Association that another attack had been made upon our present State law regulating veterinary practice. Moved and seconded, that the report be accepted; carried.

Moved and seconded, that a committee be appointed to investigate the legality of the appointment of laymen as meat-inspectors, with full power to act; carried. President Robertson thereupon appointed the following committee to act in that capacity: Dr. H. D. Hanson, chairman; H. D. Gill, C. E. Clayton.

The following committees were also appointed by the President: Arthur O'Shea Testimonial—Dr. J. E. Ryder, chairman; Roscoe R. Bell, T. Delaney. Prosecuting Committee—Dr. F. C. Grenside, chairman; H. D. Gill, E. B. Ackerman.

The following visitors were present: Dr. Bertram, Newport, R. I.; Dr. Walker and Thomas E. Mannix, Brooklyn, and students from the veterinary colleges.

Meeting adjourned at 11 P.M.

ROBERT W. ELLIS, D.V.S.,
Secretary.

### MANITOBA VETERINARY ASSOCIATION.

AT a recent meeting of this Association the following officers were elected: President, H. D. Smith, Winnipeg; Vice-President, John Spiers, Virden; Treasurer and Registrar, W. J. Hinman, Winnipeg. Council: H. D. Smith, J. Spiers, A. E. Williamson, J. H. Lipsett, M. B. Rombaugh, W. S. Henderson, and W. J. Hinman.

The Association also passed a strong resolution recommending the tuberculin-test and advocating its adoption by stockbreeders. This was transmitted to the various breeders' associations then in session and unanimously adopted by them.

